# Comparative Strength Study of Indirect Permanent Restorations: 3D-Printed, Milled, and Conventional Dental Composites

**DOI:** 10.3390/clinpract14050154

**Published:** 2024-09-20

**Authors:** João Paulo Mendes Tribst, Adelheid Veerman, Gabriel Kalil Rocha Pereira, Cornelis Johannes Kleverlaan, Amanda Maria de Oliveira Dal Piva

**Affiliations:** 1 Department of Reconstructive Oral Care, Academic Centre for Dentistry Amsterdam (ACTA), Universiteit van Amsterdam and Vrije Universiteit, 1081LA Amsterdam, North Holland, The Netherlands; 2 Department of Restorative Dentistry, Faculty of Dentistry, Federal University of Santa Maria (UFSM), Santa Maria 97105900, Rio Grande do Sul State, Brazil; gabriel.pereira@ufsm.br; 3 Department of Dental Materials Science, Academic Centre for Dentistry Amsterdam (ACTA), Universiteit van Amsterdam and Vrije Universiteit, 1081LA Amsterdam, North Holland, The Netherlands; c.kleverlaan@acta.nl (C.J.K.); a.m.de.oliveira.dal.piva@acta.nl (A.M.d.O.D.P.)

**Keywords:** 3D printing, indirect composite restorations, fracture load, finite element analysis

## Abstract

**Background/Objectives**: Limited research has been performed to assess the strength of resin-bonded 3D-printed restorations. Based on that, this study investigates the impact of different manufacturing methods on the fracture load of indirect composite restorations (ICRs) following an aging process. **Methods**: Three manufacturing techniques—conventional (CRC), milled (MRC), and printed (PRC)—were evaluated using 60 specimens, each with a diameter of 10 mm and a thickness of 1.0 mm. Sandblasting with Al_2_O_3_ particles was employed to optimize the bonding process, significantly influencing surface roughness parameters (Ra, Rz, RSm). All specimens were bonded to the dentin analog using composite resin cement and subjected to either 10,000 thermocycles (TC) or storage (ST) at 37 °C in distilled water. Fracture load assessments were performed using a universal testing machine. A finite element analysis was conducted to assess stress distribution. **Results**: Two-way ANOVA results indicated that the manufacturing method significantly affected mean fracture load values (*p* < 0.001), with PRC showing the highest mean fracture load (4185 ± 914 N), followed by MRC (2495 ± 941 N) and CRC (599 ± 292 N). The aging protocol did not have a significant impact on fracture load. **Conclusions**: This study revealed that 3D-printed resin composite exhibited comparable strength to milled resin composite when adhesively cemented, suggesting it is a promising option for indirect composite restorations based on its mechanical performance. However, further research is needed to evaluate its bond strength and optimal surface treatment methods to prevent early debonding.

## 1. Introduction

In dentistry, three main techniques are used to manufacture indirect composite restorations: direct, semi-direct, and indirect. Within these techniques, three production methods are available: conventional (CRC), milled (MRC), and printed (PRC). The quantity, scope, and location of the restoration determine the most appropriate technique for a given case [1]. The use of direct composite resin as a permanent dental restorative material has significantly increased in recent years due to its aesthetic appeal, conservative preparation, and ability to bond to tooth structure [2]. However, polymerization shrinkage during light curing is a known cause of adhesive and cohesive failures [3,4].

The indirect technique offers excellent occlusal and interproximal anatomy in large cavities. Additionally, due to the indirect nature of their production process, the polymerization shrinkage is restricted to the thin layer of resin cement, leading to reduced polymerization stress between the restoration and the tooth in comparison with direct techniques [5]. However, indirect restorations require at least two appointments: one for preparation and another for final placement. The semi-direct technique combines elements of both direct and indirect approaches, where the resin is shaped in a model, light-cured, removed for extra-oral finishing and polishing, and then adhesively cemented [6].

Although composite resin materials used for semi-direct and indirect techniques may share similar components and behavior, they can be processed differently. Today, it is possible to produce permanent indirect composite restorations using the traditional incremental technique or digital workflow with the aid of Computer-Aided Design/Computer-Aided Manufacturing (CAD/CAM) through milling or three-dimensional (3D) printing.

Tetric CAD (Ivoclar, Schaan, Liechtenstein) is an example of a CAD/CAM resin block used for permanent milled resin composite (MRC) restorations. Unlike conventional resin composites, MRCs do not undergo polymerization shrinkage within the tooth, reducing stress and the potential for leakage [7]. Indirect restorations in MRC offer better contouring of proximal surfaces, improved fracture resistance, and enhanced biocompatibility. However, these benefits come with increased costs and time, and MRCs also present a low potential for repair [7]. In contrast, Formlabs GmbH (Berlin, Germany) produces a 3D-printable composite resin (PRC) for manufacturing permanent restorations (Permanent Crown Resin). Since MRC production can result in up to 70% of material waste, 3D printing might represent a more sustainable method for producing indirect composite restorations (ICRs) [8]. However, information about the mechanical properties of PRCs is limited, raising concerns about their long-term durability and reliability compared to materials used in established techniques [8,9].

Clearfill AP-X (Kuraray Noritake Dental Inc., Tokyo, Japan) is an example of a conventional micro-hybrid resin composite (CRC) used for direct and semi-direct restorations via the incremental technique. It is known for its aesthetic and acceptable mechanical properties, such as wear resistance and the ability to bond to tooth structure [10,11]. Due to the presence of bisphenol A-glycidyl methacrylate in its composition, Clearfill AP-X exhibits high viscosity, low volatility, and reduced polymerization shrinkage [12]. However, low-certainty evidence suggests a much higher risk of secondary caries and almost double the failure rate compared to amalgam restorations [13].

One important factor for the longevity of composite restorations is their ability to withstand masticatory forces [14]. In particular, the fracture load of restoration is a critical measure of its strength and ability to resist stress [14]. Previous studies have investigated the mechanical properties of composite resins, including their flexural strength, hardness, and wear resistance [8,15,16,17], and long-term clinical studies have shown acceptable performance [15]. Despite that, the mechanical response of the bonded restoration is not always predictable solely based on the restoration stiffness, as the processing method and aging can also play a significant role [18].

While numerous studies have evaluated the fracture load of milled composite resin restorations, limited research has focused on the fracture load of 3D-printed restorations [19,20,21]. Given the increasing use of indirect composite restorations in clinical practice, it is essential to understand how different manufacturing methods contribute to the bonded fracture load and to identify potential areas for improvement. Therefore, this study aimed to evaluate the impact of manufacturing techniques on the fracture load of composite resin restorations after aging. The null hypothesis adopted was that there would be no difference in the fracture load of indirect resin composite restorations manufactured using different techniques, regardless of the aging procedure.

## 2. Materials and Methods

This study’s design was approved by the ethical commission of ACTA (2023-50219). The exact components for each material were described in the manufacturers’ manuals and are shown in Table 1.

### 2.1. Specimen’s Preparation

#### 2.1.1. Resin Disc Preparation

Resin composite discs were prepared according to the following procedures:

- Conventional resin composite (CRC): To produce the direct resin composite discs (*n* = 20) a matrix has been used, which had an inner diameter of 10 mm and a thickness of 1.0 mm. The matrix was placed on a glass plate and filled with the composite resin Clearfill APX, according to the incremental technique. Another glass plate was pressed on top of the matrix with standardized weight and the resin composite was light-cured for 20 s, according to the manufacturer’s instructions.
- CAD/CAM resin composite (MRC): For the milled specimens (*n* = 20), a Tetric CAD block was firstly drilled in a 10 mm cylinder and then sliced into samples with a thickness of approximately 1.0 mm using a Precision saw (Buehler, Isomet 1000).
- Printed resin composite (PRC): The 3D-printed specimens (*n* = 20) have been manufactured by Formlabs GmbH company according to the standard parameters provided by them. The Permanent Crown Resin, a tooth-colored, ceramic-filled resin for 3D printing of permanent single crowns, inlays, onlays, and veneers, was used.

All discs were polished by grinding using wet silicon carbide papers (SiC, 400-grit, and 600-grit papers) until a thickness of 1.0 mm using a grinding machine (Ecomet, Buehler Ltd., Evanston, IL, USA). Finally, all samples (*n* = 60) were cleaned with ethanol in an ultrasonic bath, and air surface defects were checked. Diameter and thickness were checked using a digital caliper (Absolute Digimatic, Mitutoyo, Tokyo, Kanto, Japan). Defective specimens were excluded from this study (*n* = 3) and replaced with new ones.

#### 2.1.2. Surface Treatment

All discs (*n* = 60) were sandblasted (P-G 400, Harnisch+Rieth, Winterbach, Germany) with Al_2_O_3_, 50 μm, at 1.5 bar, for 10 s, and at 10 mm distance for an optimal bonding procedure, as described in the manufacturers’ manuals. After sandblasting, the sample was cleaned with ethanol in an ultrasonic bath for 5 min, dried with compressed air, and numbered on the non-tested side with a permanent marker before testing the surface roughness.

#### 2.1.3. Surface Roughness Assessment 

All resin discs’ surface roughness was investigated through 3 measurements performed in 3 random different areas at a speed of 0.2 mm/s by a contact profilometer (SJ-400, Mitutoyo, Tokyo, Kanto, Japan) [22], both before and after sandblasting. Conditions were established in the machine, such as stand (ISO’97), Filter (Gauss), Length (2.0 mm), and Range (800). This study used 3 parameters: Ra, Rz, and RSm (in μm). Ra is the average roughness height measured from the mean line, often used for quality control. Rz is the maximum roughness depth, preferred for evaluating sealing or coating efficiency. RSm is a parameter that represents the mean spacing between the surface irregularities, useful for analyzing wear patterns and lubrication properties [23].

#### 2.1.4. Cementation Procedure

As a dentin analog, epoxy resin filled with glass fibers (NEMA grade 10, Carbotec GmbH & Co. KG, Mülheim an der Ruhr, Germany) has been considered due to its physical, chemical, and mechanical properties that are similar to those of humid dentin [24,25]. The substrate discs (*n* = 60) with a 2.0 mm thickness and a 10 mm diameter were produced out of a plate, using a consistent water-cooled drill.

After, all discs (*n* = 60) were polished by using wet silicon carbide papers (SiC, 400-grit papers) and were checked on diameter and thickness. Those that did not meet the criteria for thickness and diameter were replaced.

The dentin analogs were etched with Porcelain Etch (PE; Ultradent, Salt Lake City, UT, USA) for 1 min per sample, rinsed with water, and dried using mild compressed air. After, a tooth primer (TP; Kuraray Noritake Dental, Tokyo, Kanto, Japan) was applied to all dentin analogues for 20 s, before thoroughly drying with mild compressed air [26].

After, all resin discs were pre-processed with Clearfill Ceramic Primer (CCP; Kuraray Noritake Dental, Tokyo, Kanto, Japan) for 20 s and dried with mild air. Then, the dual resin cement Panavia V5 (Kuraray Noritake Dental, Tokyo, Japan) was applied to the resin composite discs and pressed onto the dentin analogs using a load of 4.9 N. Excess cement was removed before light curing (Bluephase PowerCure, Ivoclar, Zurich, Switzerland) the samples for 10 s per side. In total, all specimens were light-cured for 60 s. After, they were divided into 3 groups (N = 60, *n* = 20 per group) according to the material, and then they were stored in demineralized water at 37 °C for 24 h.

The final restorative set was composed of a four-layer sample, the resin composite disc on top (restoration), the cement layer, the dentin analog, and at the bottom a metal ring was attached to the set, to simulate the existence of a pulpal chamber, enabling bending of the set [27].

#### 2.1.5. Aging Protocols

Half of the specimens (*n* = 30) were submitted to 10,000 thermocycles (TC) while the other half (*n* = 30) were submitted to a storage protocol (ST) at 37 °C in distilled water (two weeks). The thermocycled (TC) groups were put in separate fully permeable plastic bags and submitted to a thermocycling machine (ACTA-TC machine, ACTA, Amsterdam, The Netherlands) (5 °C and 55 °C, 10,000 TC, 1 min/cycle), representing approximately one year of in vivo functioning [28]. To distinguish the materials during TC, each bag was marked with a specific color. 

### 2.2. Fracture Load Test

**Figure 1:**
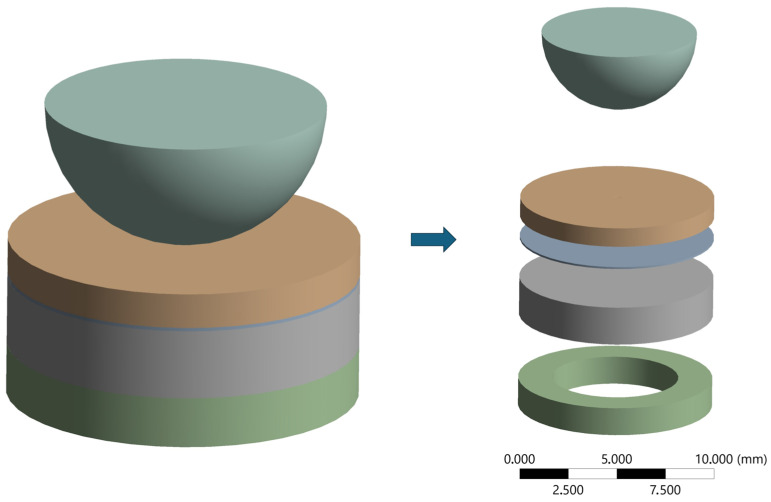
The illustrative figure of the load application over the restorative set-up (on top, the load applicator), and the 4 layers of the restorative set, on top of the resin composite disc simulating the restoration, followed by the cement layer, below that the dentin analog, and at the bottom a metal ring simulating the pulpal chamber.

After completing 10,000 TC, the fracture load (in Newtons) was measured using a universal testing machine (Instron 6022, Norwood, MA, USA) with a 10 kN load cell at a linear speed of 0.5 mm/min using the piston-on-ring test set. The load to failure was captured at the sign of a crack, and debonded samples (*n* = 4) were excluded from the data analysis. The scheme of the tested samples is presented in Figure 1 below.

### 2.3. Finite Element Analysis

A three-dimensional (3D) finite element analysis (FEA) was conducted to determine the Maximum Principal Stress, taking into account the dimensions of the in vitro specimen and a standardized cement layer thickness of 100 µm. The geometrical models were created using computer-aided design software (Rhinoceros, version 8.0 SR8, McNeel North America) and imported in STEP data format into the analysis software (ANSYS 22.2, ANSYS Inc., Pittsburgh, PA, USA). After performing a convergence test with a 10% threshold, the mesh was formed using tetrahedral elements, and the materials’ mechanical properties were modeled assuming isotropic behavior (Table 2).

Biaxial flexural strength represents the maximum stress that a material can withstand when subjected to bending forces applied along two perpendicular directions. This value is derived through estimation rather than direct measurement, based on the material’s uniaxial strength and its anticipated performance under biaxial loading. The behavior of the specimen under bending loads is influenced by its size, shape, and aspect ratio [34]. In the present study, involving a bonded specimen, the Biaxial Flexural Strength reported is based on calculations derived from the Maximum Principal Stress, which illustrates the impact of different processing methods.

### 2.4. Statistical Analysis

Surface roughness data were evaluated with a 2-way Analysis of Variance (ANOVA) and Tukey test pairwise comparison with 95% confidence, using statistical software (Minitab 16.1.0, State College, PA, USA). For each surface roughness parameter (Ra, Rz, and RSm), restorative material and sandblasting protocol were evaluated.

Fracture load data were also evaluated with 2-way ANOVA and Tukey test for pairwise comparison with 95% confidence, using the same statistical software.

## 3. Results

### 3.1. Surface Roughness

Two-way ANOVA for each surface roughness factor is presented in Table 3, Table 4 and Table 5, while means and standard deviations (µm) of the surface roughness with and without sandblasting for all materials are shown in Table 6.

Two-way ANOVA (Table 3, Table 4 and Table 5) revealed that materials, sandblasting, and their interaction were significant for Ra, Rz, and RSm mean values (*p* < 0.001). Comparing the materials, PRC showed the highest mean value (Ra: 1.4 ± 1.0 μm; Rz: 9.7 ± 6.7 μm)^A^, followed by CRC (Ra: 1.3 ± 1.1 μm; Rz: 8.5 ± 6.9 μm)^B^ and MRC (Ra: 1.0 ± 0.9 μm; Rz: 7.2 ± 6.1 μm)^C^. In addition, sandblasted specimens were rougher (Ra: 2.3 ± 0.2 μm; Rz: 14.8 ± 1.5 μm)^A^ than the specimens without this treatment (Ra: 0.2 ± 0.1 μm; Rz: 2.1 ± 0.9 μm)^B^. Table 6 revealed that after sandblasting, both PRC and CRC showed similar and higher Ra and Rz surface roughness.

For RSm, CRC (RSm: 77.7 ± 41.6 μm)^B^ and MRC (RSm: 76.7 ± 33.1 μm)^C^ showed a nonsignificance, contrary to PCR (RSm: 99.2 ± 38.2 μm)^A^ which showed a significant difference.

### 3.2. Fracture Load

**Figure 2:**
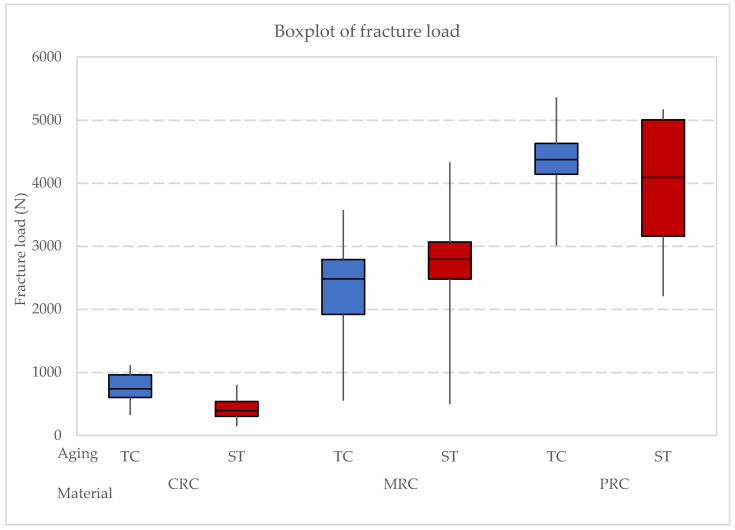
Boxplot of fracture load according to the resin composite (CRC: conventional resin composite, MRC: milled resin composite, and PRC: printed resin composite) and aging procedure.

Two-way ANOVA (Table 7) revealed that the material was significant for fracture load mean values (*p* < 0.001) while the simulated aging protocol (*p* > 0.05) was not. Comparing the materials, PRC showed the highest mean value (4185 ± 914 N)^A^ followed by MRC (2495 ± 941 N)^B^ and CRC (599 ± 292 N)^C^. As for the aging process, storage (2461 ± 1704 N) showed slightly higher mean values than thermocycling (2327 ± 1612 N); however, the difference was not significant (*p* > 0.05). All the previously described results are shown as a boxplot (Figure 2).

One-way ANOVA was performed considering both factors together (material and aging procedure) which revealed a significant difference in the load to failure between the groups (*p* < 0.001) (Table 8). Means (N) and standard deviations (StDev) of load to failure are shown in Table 9, as well as the Tukey test distribution.

**Figure 3:**
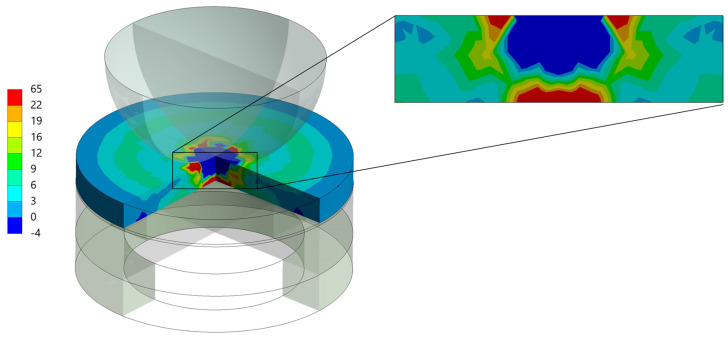
Representative images of the finite element analysis (FEA) simulating stress concentration at the mean fatigue failure load observed for each restorative setup during loading.

The load-to-failure data utilized in the finite element analysis are outlined in Table 10. Figure 3 depicts the peak tensile stress (Maximum Principal Stress) observed in the restoration model following the results’ analysis. This figure shows where stress is most concentrated at the specimen’s bottom, correlating with the areas of failure.

## 4. Discussion

This study evaluated how different manufacturing techniques affect the fracture load of indirect composite restorations after simulated aging. The findings indicate that sandblasting the surface of the specimens as a pre-treatment before cementation increases surface roughness, thereby preparing the surface for bonding. Moreover, significant differences in fracture load among the materials were observed, regardless of the aging protocol, leading to the rejection of the study’s null hypothesis. This finding confirms the different performance between materials and the lack of influence of the aging protocols.

According to the manufacturer, the evaluated milled composite (MRC) has a fracture load of 2600 N, which aligns with the data from this study. It was anticipated that MRC would exhibit the highest fracture load, followed by conventional resin composite (CRC) and then by printed resin composite (PRC), based on their reported flexural strength values. Contrary to expectations, PRC demonstrated the highest fracture load, followed by MRC and CRC. This might be partially explained by the effects of sandblasting, which increases surface roughness. Laboratory investigations suggest that rough surfaces can negatively impact mechanical characteristics [35,36]. Research by Yoshihara et al. [37] indicates that minor surface imperfections on roughened surfaces may develop into initial cracks under compressive loads, leading to catastrophic failure due to non-uniform stress distribution [38,39,40]. This explains the decreased fracture load observed in CRC and MRC. However, the high fracture load of PRC, despite its increased surface roughness, requires further investigation. In addition, finite element analysis (FEA) suggests that while the stress magnitude in the restoration was close to the manufacturer’s reported strength, the stress in the cement layer was very high in MRC and PRC, indicating potential debonding before material fracture.

Despite not showing a significant difference between aging protocols, it is noteworthy that 96.7% of CRC specimens survived water storage, and 90% of specimens subjected to thermocycling also survived. CRC did not experience any bonding failures after thermocycling, while MRC and PRC had one and three debonded specimens, respectively. The debonding in PRCs could be attributed to polymerization stress or lack of sufficient bond strength. However, no literature is available on the polymerization rate of this or similar PRC materials, suggesting a need for further research.

Temperature fluctuations and complex chewing pressures in the oral cavity significantly impact the flexural stress in composite restorations, leading to material fatigue [41,42]. This underscores the importance of simulating fatigue in studies of dental restoration materials. Water storage and thermocycling are standard procedures in dental research to simulate aging. Thermocycling, which involves exposing materials to extreme temperature changes over many cycles, is more indicative of real-life conditions than water storage [43]. Studies have shown that thermocycling can reduce flexural strength and microhardness [16,44,45], although some research, like that by de Oliveira et al. [46], suggests that micro-hybrid resin composites maintain their hardness value despite thermocycling. This study did not observe significant changes in fracture load after water storage and 10,000 thermal cycles. According to Henderson et al. [47], further research is needed to fully understand the effects of thermocycling on fracture load.

A recent scoping review highlighted that while 3D-printed composites hold promise, they currently fall short in hardness, flexural strength, and overall mechanical durability compared to traditional materials. Their performance as long-term permanent restorations remains inadequate [18,48]. Nevertheless, this study shows that 3D-printed composites can be viable for indirect restorations when properly cemented. Rosentritt et al. [49] found that printed and milled permanent crowns exhibited acceptable mid-term performance and wear stability, with similar fracture forces. However, their study did not confirm that PRC had superior fracture forces compared to MRC.

Considering the dentin analog used in this study, it is important to acknowledge that no material perfectly replicates natural dentin. Epoxy filled with glass fibers (NEMA grade 10) is considered a suitable dentin analog, although it may have some differences [50]. These differences are generally minor and may not significantly impact the performance of dental restorations [50]. Thus, further exploration of factors such as flexural strength, water sorption, and material wear is essential for improving the longevity and reliability of indirect resin composite restorations. Enhanced understanding of fracture load can lead to the development of more effective materials and techniques for testing dental restorations.

In summary, the present study demonstrates the significant influence of manufacturing techniques on the fracture load of indirect composite restorations (ICRs), with 3D-printed resin composites (PRC) exhibiting adequate behavior. These findings align with previous research on the mechanical properties of 3D-printed dental materials, which highlighted the promising performance of high-filled 3D-printed resins [51]. Interestingly, aging protocols, such as thermocycling and water storage, did not significantly impact fracture load in any group, suggesting the resilience of PRC under simulated oral conditions. These findings are consistent with studies evaluating the durability of 3D-printed restorations, which have shown that post-processing conditions like thermal aging may not critically compromise their mechanical integrity [52,53]. Additionally, finite element analysis reinforced the superior stress distribution in PRC, possibly contributing to its higher load-bearing capacity. These results suggest that 3D printing offers a viable alternative to conventional and milled methods, justifying further investigation into bond strength and surface treatment optimization for long-term clinical success [52,53,54].

## 5. Conclusions

This study revealed that adhesively bonded 3D-printed resin composite exhibited comparable strength to milled resin composite, suggesting it is a promising option for indirect composite restorations based on its mechanical performance. However, further research is needed to evaluate its bond strength and optimal surface treatment methods to prevent early debonding.

## Figures and Tables

**Table 1:** Material, manufacturer, components, and flexural strength.

Manufacturing Method	Resin Composite, Manufacturer	Components ^1^	Flexural Strength (MPa) ^1^
Conventional Resin Composite (CRC)	Clearfill APX A4, Kuraray	Silanated barium glass fiber Bisphenol A-diglycidyl methacrylate (Bis-GMA), Silanated colloidal silica, Triethyleneglycol dimethacrylate (TEGDMA), Silanated silica filler dl-Camphorguinone, Inorganic filler approx. 71 vol%, Particle size of inorganic filler 0.02 to 17 μm.	204
Milled Resin Composite (MRC)	Tetric CAD Cerec/inLab HT (C14) A3, Ivoclar	Bisphenol A-diglycidyl dimethacrylate (Bis-GMA), Ethoxylated bisphenol A dimethacrylate (BIS-EMA), Triethylene glycol dimethacrylate (TEGDMA), Urethane dimethacrylate (UDMA), Barium glass fillers and silicon dioxide fillers, Total filler volume approx. 51 vol%, Additives and pigments.	273.8
Printed Resin Composite (PRC)	3D-printed permanent crown A2, Formlabs GmbH	4,4′-isopropylideendifenol ethoxylated, 2-methylprop-2-enoicacid, Difenyl(2,4,6-trimethylbenzoyl) fosfineoxide.	116

^1^ According to the manufacturers.

**Table 2:** Materials properties are based on the literature.

Material	Elastic Modulus (GPa)	Poisson Ratio	Reference
Conventional Resin Composite (CRC)	16.8	0.3	[29]
Milled Resin Composite (MRC)	14.0	0.3	[30]
Printed Resin Composite (PRC)	4.26	0.3	[31]
Dentin analogue	18.6	0.3	[32]
Cement	6.0	0.3	[33]

**Table 3:** Two-way analysis of variance for Ra parameter according to material and sandblasting protocol. *: It means the interaction between two factors.

Source	DF	Adj SS	Adj MS	F-Value	*p*-Value
Material	2	2.952	1.476	48.02	<0.001
Sandblasting	1	131.106	131.106	4265.13	<0.001
Material * Sandblasting	2	0.687	0.344	11.18	<0.001
Error	114	3.504	0.031		
Total	119	138.249			

**Table 4:** Two-way analysis of variance for Rz parameter according to material and sandblasting protocol. *: It means the interaction between two factors.

Source	DF	Adj SS	Adj MS	F-Value	*p*-Value
Material	2	114.46	57.23	39.96	<0.001
Sandblasting	1	4905.39	4905.39	3424.98	<0.001
Material * Sandblasting	2	15.22	7.61	5.31	0.006
Error	114	163.28	1.43		
Total	119	5198.34			

**Table 5:** Two-way analysis of variance for RSm parameter according to material and sandblasting protocol. *: It means the interaction between two factors.

Source	DF	Adj SS	Adj MS	F-Value	*p*-Value
Material	2	12,524	6261.8	10.41	<0.001
Sandblasting	1	88,609	88,608.6	147.26	<0.001
Material * Sandblasting	2	6256	3128.2	5.20	0.007
Error	114	68,597	601.7		
Total	119	175,986			

**Table 6:** Mean (μm) and standard deviation (StDev) of Ra, Rz, and RSm surface roughness parameters.

Resin Composite	Sandblasting	Roughness Parameters (Mean ± StDev) ^1^		
		Ra	Rz	RSm
CRC	No	0.1 ± 0.0 ^D^	1.8 ± 0.3 ^D^	42.3 ± 15.1 ^C^
	Yes	2.4 ± 0.1 ^A^	15.4 ± 1.5 ^A^	115.3 ± 16.3 ^A^
MRC	No	0.2 ± 0.0 ^D^	1.3 ± 0.6 ^D^	51.7 ± 26.3 ^C^
	Yes	2.0 ± 0.2 ^B^	13.2 ± 1.5 ^B^	101.8 ± 15.5 ^AB^
PRC	No	0.4 ± 0.1 ^C^	3.2 ± 1.0 ^C^	79.6 ± 43.0 ^B^
	Yes	2.5 ± 0.2 ^A^	16.1 ± 1.6 ^A^	118.8 ± 18.5 ^A^

^1^ Mean values in the same column that do not share a letter represent a significant difference.

**Table 7:** Two-way ANOVA for fracture load according to material and aging factors. *: It means the interaction between two factors.

Source	DF	Adj SS	Adj MS	F-Value	*p*-Value
Material	2	118,218,794	59,109,397	99.30	<0.001
Aging	1	61,547	61,547	0.10	0.749
Material * Aging	2	1,863,727	931,863	1.57	0.219
Error	50	29,764,383	595,288		
Total	55	149,091,230			

**Table 8:** One-way ANOVA for load to failure.

Source	DF	Adj SS	Adj MS	F-Value	*p*-Value
Group	5	119,326,848	23,865,370	40.09	<0.001
Error	50	29,764,383	595,288		
Total	55	149,091,230			

**Table 9:** Fracture load mean values ± standard deviation (StDev) according to the material and aging protocol. ^A–C^: Mean values in the same column that do not share a letter represent a significant difference.

Group	Resin Composite	Aging Protocol	Mean ± StDev
CRCtc	Conventional Resin Composite	Thermocycling	760 ± 230 ^C^
CRCst		Storage	439 ± 264 ^C^
MRCtc	Milled Resin Composite	Thermocycling	2271 ± 886 ^B^
MRCst		Storage	2719 ± 983 ^B^
PRCtc	Printed Resin Composite	Thermocycling	4348 ± 693 ^A^
PRCst		Storage	4021 ± 1073 ^A^

**Table 10:** Maximum Principal Stress (MPa) according to the material and aging protocol for the restoration and cement layer.

Group	Resin Composite	Aging Protocol	Restoration	Cement Layer
CRCtc	Conventional Resin Composite	Thermocycling	32.10	7.33
CRCst		Storage	45.13	14.49
MRCtc	Milled Resin Composite	Thermocycling	127.83	66.64
MRCst		Storage	151.17	81.34
PRCtc	Printed Resin Composite	Thermocycling	128.49	97.3
PRCst		Storage	118.05	90.54

## Data Availability

Data are available on reasonable request.
